# The Rival Discoverers

**Published:** 1838-04

**Authors:** 


### "THE RIVAL DISCOVERERS."

We recommend to the attention of our readers an excellent paper with this heading, which appeared in a late number of the Medical Gazette, relating to a subject which we also find ourselves called upon, as journalists, to notice. On more than one occasion, since the commencement of our critical labours, have we felt ourselves under the necessity of animadverting, in somewhat strong terms, on the very unphilosophical temper in which some members of our profession set about their scientific investigations, and particularly, on the envious and uncharitable spirit with which certain physiologists are accustomed to assert their rival claims of discovery. "Such (we have said) are the fretful labours of controversialists who allow the love of fame to predominate over the pure love of truth. Their restless obtrusion of their real or supposed discoveries, their fidgety fear of being run over and forgotten, their challenges and reclamations, their attacks and replies and rejoinders, whilst they doubtless disqualify them for being sound sleepers, neither amuse nor instruct nor convince, but make the paths of science thorny and unpleasant, which should be full of peace and delight." . . . "Thus mortal men, using the intellect which God has given them, make ever slow advances along the boundaries of knowledge, sometimes in column, and sometimes in equal line of march; and happy is it when, on lighting on some undiscovered truth, they do not turn round or aside, and contend with their followers or fellows, as is the wont of carnivorous creatures over their precarious food, disgracing the understanding and mocking the dignity of the human creature by the passions of the lower tribes of animals. It was a saying of Sir James Mackintosh, that, when asked in another world what he had been doing all his lifetime in this, he should have no better answer to make than that 'he had been talking.' Of how many a physiologist the reply must be—'I have been fighting:' not from the calmness and silence of the study, with unwrinkled brow advancing, but with flushed cheeks and soul-marks of intellectual blows."

Since these strictures appeared in our last Number, our literature and our science have been alike disgraced by an exhibition of the kind referred to, and from the same quarter, but much more outrageous than any that has preceded it. The readers of the weekly Journals will be at no loss to know that we allude to the recent controversy between Drs. Grant and Hall and Mr. Newport, touching their respective claims to certain discoveries, and to the promulgation of certain opinions, relating to the nervous system of insects. Of the nature of these claims we shall take another occasion to speak, and shall endeavour to award impartial justice to the contending parties, forming our judgment from the coolest and most careful examination of the evidence produced. But the question of claims so fiercely contended for by the parties immediately interested, and even the correctness of our own decision, (so long as we have the consciousness of knowing that we wished to decide correctly,) are to us matters of little moment in comparison with the injury which has been inflicted on the character of our profession by the manner in which the controversy has been conducted. In the position in which we are placed, as one of the public organs of the profession in this country, we hold it to be our duty, in vindication of the honour of that profession, to declare that its members, as a body, hold no sympathy with the low and evil passions disclosed in the communications of Dr. Grant and Dr. Hall, and repudiate the employment, in scientific warfare, of the weapons of mere obloquy and personal invective, so fiercely and lavishly wielded by them against Mr. Newport. Had every one of the charges of delinquency brought against this gentleman been true, instead of being, as he maintains, utterly groundless, and the offspring of misapprehension or malevolence; although we might, in this case, have made allowance for much of the severity of the language used by Dr. Grant and Dr. Hall, it would have been impossible to justify or excuse, or even to regard without disgust, the detail of private circumstances intruded into the controversy by the last-named physician. Often before now has it happened,—and often, we trust, it will again happen,—that men of science have mutually given and received assistance, both personal and pecuniary; but we hardly think that it ever before occurred, among men in the upper ranks of society, that such pitiful and paltry obligations as those conferred on Mr. Newport by Dr. Hall, were trumpeted forth to the world by the individual himself, and with all the pomp and circumstance of offended patronage. We will not disfigure our pages by enumerating them. It is no wonder that Mr. Newport should say, "Surely there never was such a practical sophistry attempted to be maintained, and hardly ever was there put on record charges more unjust or more insidious;" or that he should add, "I know not how my statement may be received by Dr. Hall, but I shall think much worse of him than I now do, if he feels no remorse on reading the details he compels me to publish; and I shall have formed a very incorrect judgment of his friends and my own, and of the members of the profession generally, if the history of our acquaintance is not considered to be at least as humiliating to him as to myself." We think the circumstances disclosed in no degree humiliating to Mr. Newport; but we are greatly mistaken if Dr. Hall will not ere long make the discovery,—a discovery which all that he has already made or imagined will not lightly counterpoise,—that, on the present occasion, he has allowed himself to be betrayed by his passions into a forgetfulness of his own dignity and self-respect, and that he has sustained, in consequence, a proportionate loss in the estimation of his friends.

